# Evaluating Machine Learning Models for Predicting Late Leprosy Diagnosis by Physical Disability Grade in Brazil (2018–2022)

**DOI:** 10.3390/tropicalmed10050131

**Published:** 2025-05-12

**Authors:** Lucia Rolim Santana de Freitas, José Antônio Oliveira de Freitas, Gerson Oliveira Penna, Elisabeth Carmen Duarte

**Affiliations:** 1 Faculdade de Medicina, Universidade de Brasília, Brasília 70910-900, Brazil; eduarte@unb.br; 2 Departamento de Matemática, Universidade de Brasília, Brasília 70910-900, Brazil; jfreitas@unb.br; 3 Escola de Governo Fiocruz Brasília, Fundação Oswaldo Cruz, Brasília 70904-130, Brazil; gerson.penna@fiocruz.br; 4 Núcleo de Medicina Tropical, Universidade de Brasília, Brasília 70910-900, Brazil

**Keywords:** leprosy, risk factors, disability, neglected diseases, machine learning, epidemiology

## Abstract

The severity of physical disability at leprosy diagnosis reflects the timeliness of case detection and the effectiveness of disease surveillance. This study evaluates machine learning models to predict factors associated with late leprosy diagnosis—defined as grade 2 physical disability (G2D)—in Brazil from 2018 to 2022. Using an observational cross-sectional design, we analyzed data from the Notifiable Diseases Information System and trained four machine learning models: Random Forest, LightGBM, CatBoost, XGBoost, and an Ensemble model. Model performance was assessed through accuracy, area under the receiver operating characteristic curve (AUC-ROC), recall, precision, F1 score, specificity, and Matthew’s correlation coefficient (MCC). An increasing trend in G2D prevalence was observed, averaging 11.6% over the study period and rising to 13.1% in 2022. The Ensemble model and LightGBM demonstrated the highest predictive performance, particularly in the north and northeast regions (accuracy: 0.85, AUC-ROC: 0.93, recall: 0.90, F1 score: 0.83, MCC: 0.70), with similar results in other regions. Key predictors of G2D included the number of nerves affected, clinical form, education level, and case detection mode. These findings underscore the potential of machine learning to enhance early detection strategies and reduce the burden of disability in leprosy, particularly in regions with persistent health disparities.

## 1. Introduction

Leprosy is a chronic granulomatous infection commonly caused by *Mycobacterium leprae* and *Mycobacterium lepromatosis*, whose incidence and prognosis are associated with high social vulnerability and poor access to health services [1,2,3,4]. Although it is curable and can be controlled as a public health problem [5], addressing leprosy and its consequences remains a major challenge in many parts of the world [6,7].

In 2023, the World Health Organization (WHO) reported 182,815 new cases of leprosy worldwide, 24,773 of which were recorded in the Americas, with approximately 22,773 (91.9%) new cases reported in Brazil. Of these patients, 2173 (9.5%) already had significant impairment—grade 2 physical disability (G2D)—at the time of diagnosis [8].

Leprosy is diagnosed based on epidemiological and clinical signs and symptoms, such as skin lesions with sensory disturbance, supported by complementary laboratory tests. Without an assertive and timely diagnosis and appropriate clinical management, the disease can evolve with neurological impairment and sequelae due to the involvement of peripheral nerves, disabilities, deformities, and stigmas.

The degree of physical disability at the time of diagnosis is an important factor associated with neurological deterioration before treatment [9,10]. Therefore, at the time of diagnosis, the WHO has developed and recommended the use of a system to monitor the severity of leprosy-related physical disability as a marker of early case detection, the quality of disease surveillance, and the burden of disease in a population [11,12].

Despite significant advancements in epidemiological surveillance [13], there is still a lack of precise, data-driven methodologies for predicting late leprosy diagnosis. While conventional statistical models have identified risk factors associated with delayed detection, their predictive power remains limited. Recent advances in machine learning (ML) have demonstrated significant potential in addressing public health challenges, including the prediction of disease outcomes and the potential of ML in improving disease classification and risk stratification for infectious diseases [14,15,16]. However, the application of ML models for predicting late leprosy diagnosis based on disability grading remains underexplored. This study aims to evaluate the performance of machine learning models in predicting late leprosy diagnosis, as measured by G2D at diagnosis, in Brazil from 2018 to 2022.

## 2. Materials and Methods

This analytical cross-sectional study used data from the Notifiable Diseases Information System (SINAN) [17]. The study population consisted of new leprosy cases notified in the period 2018 and 2022.

The variables analyzed were age group (in years: <15, 15–29, 30–49, 50–69, 70–79, 80 and over), sex, race or skin color (white, yellow, brown, and black), schooling (higher education incomplete or complete, high school incomplete or complete, 8th grade complete or 5th to 8th grade incomplete, 1st to 4th grade incomplete or complete, illiterate ignored and not applicable (under 7 years old)), number of nerves affected (0, 1, 2 to 5, and >5), clinical form (indeterminate, tuberculoid, virchowian, diforma, and unclassified), operational classification (paucibacillary or multibacillary), method of case detection (referral, spontaneous demand, collective examination, examination of contacts, other methods, and unknown), year of notification, degree of disability at the time of diagnosis (0, 1, and 2, according to WHO classification 1988 [11,18]).

The high incapacitating potential of the disease can be verified by the degree of physical incapacity (GIF), which is determined from neurological assessments of patients’ eyes, hands, and feet. The scores are as follows: GIF 0, when muscle strength and sensitivity of these segments are preserved; GIF 1, when there is a decrease in muscle strength and/or sensitivity; and GIF 2, when visible disabilities, deformities in the hands, feet, and/or eyes are observed [19]. The outcome variable G2D, representing grade 2 disability, was classified into two categories: G2D = yes (GIF = 2) and G2D = no (GIF = 0/1). In this study, G2D is the target variable, and we aim to predict its occurrence using the explanatory variables presented before.

Descriptive statistics were used to summarize the characteristics of the study population. Categorical predictors were presented as frequencies and percentages. Differences between G2D were tested using the Chi-square test for categorical predictors. All qualitative predictors were handled through one-hot encoding, where each category was considered separately for this procedure. We tested four different ML algorithms: Random Forest, CatBoost, XGBoost, LightGBM, and an ensemble of the four models. For CatBoost, XGBoost, and LightGBM, we used their respective Python packages. For the other algorithms, we used the scikit-learn library. Additionally, we employed the bootstrapping technique to further ensure the robustness and reliability of the model’s performance.

To ensure generalizability and prevent overfitting, the dataset was divided into training and test sets in an 80:20 ratio using stratified random sampling to maintain the same proportion of the outcome variable. Each region (north, northeast, southeast, south, and midwest) was proportionally represented in both subsets. All predictor variables were standardized to ensure uniformity across different scales. Machine learning models were trained using a 5-fold cross-validation approach, where the dataset was divided into five equal folds. One fold was used for testing, while the remaining four folds were used for training. This process was repeated ten times, with each fold serving as the test set once. Each algorithm was selected based on its ability to handle structured epidemiological data and its performance in similar classification tasks. These models differ in their underlying learning mechanisms and suitability for this analysis [20,21]. Hyperparameter selection was performed using a randomized search in the training set with 3-fold cross-validation. The Synthetic Minority Over-sampling Technique (SMOTE) was applied when the minority class represented less than 25% of the total outcomes [22]. To provide a robust assessment of model performance, we calculated the area under the receiver operating characteristic curve (AUC) for each cross-validation fold and reported the mean AUC. The evaluation of machine learning algorithms was conducted in the test set, based on metrics such as the area under the ROC curve (AUC-ROC), accuracy, precision, recall, specificity, Matthew’s correlation coefficient (MCC), and F1 score. A feature importance analysis was conducted using the permutation importance method to identify the most relevant variables contributing to the prediction of a late leprosy diagnosis.

The analyses were carried out using Anaconda Jupyter Notebook version 24.11.2, using Python version 3.12.3. This study was carried out exclusively using publicly available secondary data, without identifying the subjects, and its procedures are in accordance with the principles of ethics in research involving human beings.

## 3. Results

### 3.1. Physical Disabilities Characteristics

In Brazil, in the period from 2018 to 2022, 140,909 cases were reported, of which 128,069 (90.9%) were assessed for degree of disability at the time of diagnosis. Among these patients, 11.6% (14,793) had G2D at the time of diagnosis. This indicator shows a continuous upwards trend, from 10.0% of cases with G2D in 2018 to 13.1% in 2022.

Patients with G2D had a median age of 53 years (1st quartile = 40 years; 3rd quartile = 65 years). Of all cases notified, 14,792 (10.5%) presented with G2D at the time of leprosy diagnosis. A higher proportion of males (70.7%) exhibited G2D compared to females (29.3%). The 50–69 age group had the highest number of G2D cases (41.1%), while the 0–14 age group had the lowest (1.7%). Patients identifying as brown comprised the largest group with G2D (57.8%). Regarding education, individuals with high school (incomplete or complete) had the highest proportion of G2D (30.5%), while illiteracy was associated with a lower proportion (3.4%). A greater number of affected nerves was strongly associated with G2D, with 54.4% of those with G2D presenting two to five nerves affected, and 28% presenting more than five affected nerves. Virchowian and Diforma were the most frequent clinical forms associated with G2D, with 33.0% and 55.5%, respectively. Multibacillary cases represented the majority (96.4%) of G2D. Referral was the most common case detection mode among individuals with G2D (51.0%) (Table 1).

### 3.2. Performance of Predictive Models

The performance of four machine learning models—Random Forest, LightGBM, CatBoost, XGBoost, and an ensemble model—was evaluated across Brazil’s five regions (north, northeast, southeast, south, and midwest). All models demonstrated strong predictive capabilities, with accuracy ranging from 0.80 to 0.86 and AUC-ROC values between 0.89 and 0.94 (Table 2). The ensemble model consistently outperformed individual models, achieving the highest AUC-ROC (0.94) and F1 score (0.87) in the south region, along with the highest Mathew’s correlation coefficient (MCC) of 0.73.

In the north region, the Random Forest and LightGBM models achieved an accuracy of 0.84 and AUC-ROC of 0.92–0.93, with recall values of 0.90–0.91, indicating robust sensitivity in identifying G2D cases. Similar performance was observed in the northeast, where the ensemble model achieved an accuracy of 0.85 and AUC-ROC of 0.93. In the southeast, LightGBM and the ensemble model showed balanced performance, with an accuracy of 0.84 and AUC-ROC of 0.92. The south region exhibited the highest overall performance, with CatBoost and XGBoost achieving accuracy and AUC-ROC values of 0.86 and 0.93, respectively. The midwest region, while slightly lower in performance, still demonstrated strong predictive capabilities, with accuracy ranging from 0.80 to 0.82 and AUC-ROC values between 0.89 and 0.91. The MCC ranged between 0.60 and 0.73, suggesting robust performance even in scenarios with class imbalance.

Our results demonstrated that, in north, northeast, and midwest regions, the performance differences between the top-performing model and the others were marginal. However, in southeast and south regions, these differences were more pronounced and statistically significant, indicating that the top-performing model consistently outperformed the others in those contexts. In the north and northeast regions, where LightGBM and the ensemble model had identical performance, LightGBM was chosen due to its computational efficiency and lower complexity, making it more suitable for interpretability and reproducibility in this context.

**Figure 1:**
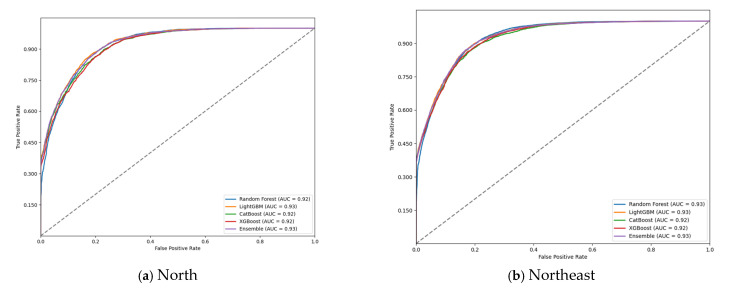

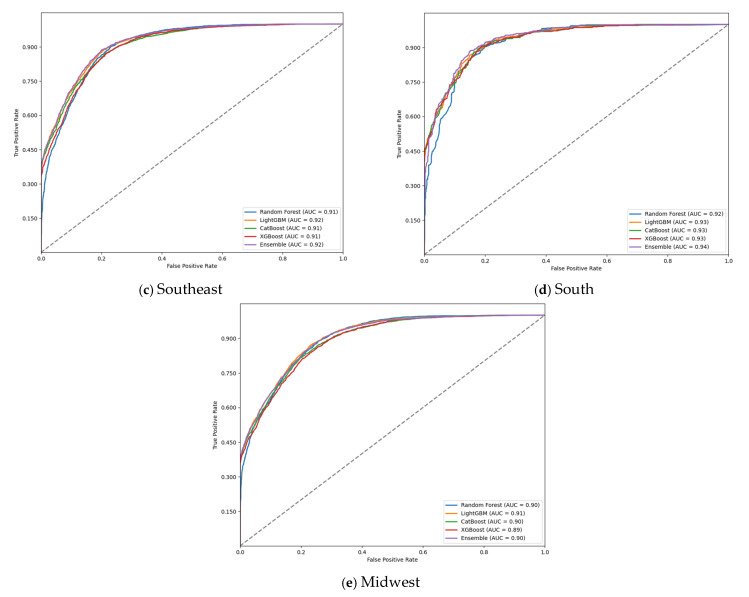
Receiver operating characteristic (ROC) curve on test data for grade 2 physical disability (G2D) prediction, according to region, 2018–2022: (**a**) north; (**b**) northeast; (**c**) southeast; (**d**) south; and (**e**) midwest.

The ROC curve evaluation of the models (Figure 1) confirmed the high discriminatory capacity of the machine learning approaches in predicting G2D. The ensemble model consistently achieved the highest AUC-ROC values across all regions, demonstrating its superior ability to distinguish between G2D and non-G2D cases. The curves also highlighted the model’s robustness, with minimal variation in performance across different geographic regions.

### 3.3. Predictor Importance

**Figure 2:**
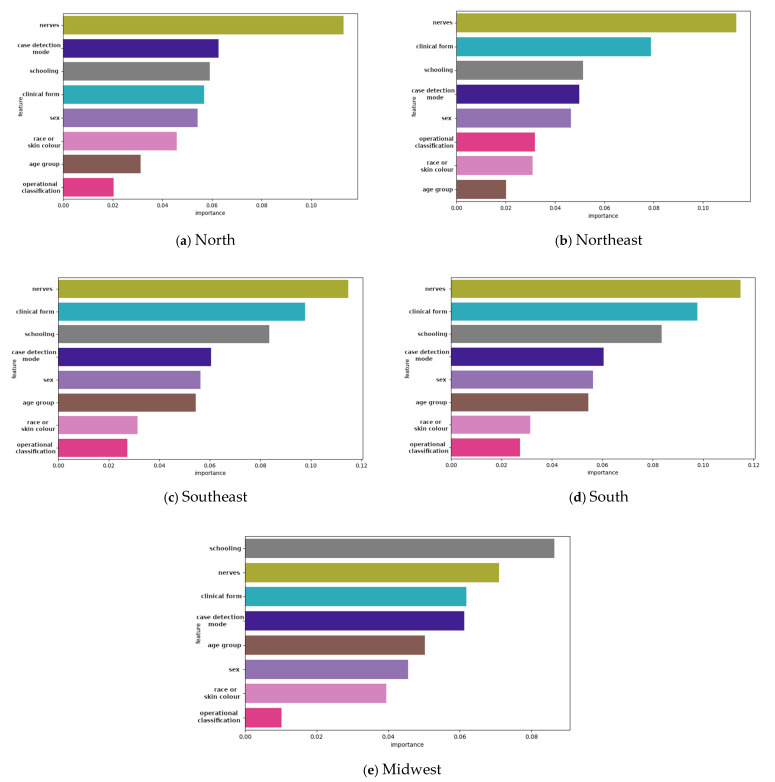
Feature importance gain plot for LightGBM algorithm and an ensemble model showing the features contributing to the model for grade 2 physical disability (G2D) according to region, 2018–2022: (**a**) north; (**b**) northeast; (**c**) southeast; (**d**) south; and (**e**) midwest.

The feature importance analysis revealed that the number of nerves affected, clinical form, and operational classification were the most significant predictors of G2D across all models. The LightGBM and ensemble models highlighted these features as having the highest gain, underscoring their critical role in predicting severe disability. Age group and education level also contributed significantly, aligning with the descriptive findings (Figure 2). The feature importance analysis revealed that the most influential predictors of G2D varied across regions. In all regions, the number of affected nerves and the clinical form of the disease were the strongest predictors. However, in the midwest region, educational level had a more pronounced impact compared to other regions.

## 4. Discussion

This study evaluated the performance of ML models in predicting G2D in leprosy cases in Brazil from 2018 to 2022, identifying key sociodemographic and clinical predictors of late diagnosis and has demonstrating robust predictive capabilities, with the ensemble model achieving the highest performance across all regions. The results demonstrate that ML-based approaches exhibit robust predictive capabilities, with the LightGBM model and ensemble model achieving the highest performance across all geographic regions. These results underscore the potential of machine learning to enhance early detection and intervention strategies for leprosy. The findings also showed a concerning trend of increasing G2D cases, with 11.6% of leprosy cases presenting severe disability at diagnosis, rising to 13.1% in 2022.

The high proportion of G2D cases at diagnosis aligns with findings from other studies in Brazil and globally. For instance, a study in Alagoas, Brazil, reported a similar G2D prevalence of 12.6% [23]. However, contrasting findings from Indonesia highlight regional disparities in diagnostic delays, with patient-related delays being more significant than health system delays [24]. These differences suggest that cultural, socioeconomic, and health system factors play a critical role in shaping leprosy outcomes.

With regard to the clinical variables of the disease, as has already been described in other studies, we have shown that the prevalence of G2D at the time of diagnosis of leprosy increases in patients with a greater number of affected nerves, with a determined clinical form, particularly virchwiana, compared to an indeterminate clinical form, and in multibacillary patients compared to paucibacillary patients [23,25,26,27,28]. Similarly, Claudino dos Santos et al. [23] reported a greater likelihood of a late diagnosis (7.6 higher; 95% CI: 4.65–12.42) in multibacillary patients than in paucibacillary patients and in patients with the tuberculoid clinical form (3.61; 95% CI 2.21–6.29) than in indeterminate patients. Differences in the strength of the associations found between the different studies, particularly between the study by Claudino dos Santos et al. [23] and ours, can be explained by methodological choices, such as the method of measuring late diagnosis (time from symptoms to diagnosis versus the presence of G2D at the time of diagnosis), the profile of the study population, and the choice of method and adjustment variables for the statistical model used.

The ML models demonstrated strong predictive capabilities for G2D, with the ensemble model achieving the highest performance across most regions. The high AUC-ROC values across all models and regions demonstrate the potential of machine learning to support early leprosy diagnosis and targeted interventions, suggesting the potential for integrating these models into public health programs. Model performance also exhibited regional differences. While the ensemble and LightGBM models consistently achieved the highest AUC-ROC values across all regions, this variation may be attributed to data quality issues, including higher rates of underreporting and misclassification, as previously reported in studies on health information system limitations in these regions [29]. In the midwest region, a Random Forest model demonstrated comparable performance to the ensemble model, suggesting that simpler models may suffice in certain contexts. This regional variation underscores the importance of tailoring ML approaches to local epidemiological profiles. Despite these challenges, the overall high discriminatory capacity of the models highlights the robustness of machine learning in predicting G2D, reinforcing its potential application in leprosy surveillance and risk stratification strategies. Furthermore, in the north and northeast regions, LightGBM and the ensemble model demonstrated identical performance. Given its computational efficiency and lower complexity, LightGBM was selected as the preferred model for these regions, enhancing interpretability and facilitating real-world implementation.

Studies evaluating ML-based predictive models for tuberculosis and neglected tropical diseases have similarly demonstrated high accuracy in identifying high-risk populations, supporting the utility of these approaches in resource-limited settings [30]. Additionally, our results corroborate prior findings on the influence of sociodemographic and clinical factors in late leprosy diagnosis, further validating the role of ML in epidemiological modeling [23,31]. However, differences in model performance across regions highlight the necessity of addressing data quality issues and ensuring that ML applications are adapted to specific healthcare system constraints.

Several studies have demonstrated that late diagnosis increases the likelihood of severe disability, reinforcing the importance of early detection and intervention [25,26,27,28,32,33,34]. A systematic review on leprosy in children under 15 years of age in Brazil revealed a concerning proportion of cases presenting with multibacillary forms and G2D at diagnosis, suggesting substantial delays in case identification [35]. Traditional epidemiological methods often rely on standard regression models, which may not fully capture complex patterns in large datasets. Advances in ML have shown promise in improving disease surveillance and the early detection of leprosy. Studies have demonstrated the potential of ML in improving early detection, risk stratification, and surveillance strategies for neglected tropical diseases, including leprosy [36,37]. For instance, Ferreira et al. (2022) [38] employed artificial neural networks to classify leprosy cases based on clinical and demographic data, outperforming traditional epidemiological models in early case identification. Rodrigues et al. (2023) [36] utilized Bayesian networks to model the occurrence of leprosy reactions, demonstrating that ML can refine risk assessment by integrating clinical and epidemiological variables. These approaches highlight the potential of ML in complementing conventional surveillance systems by identifying patterns that may be overlooked by traditional methods.

The feature importance analysis underscored the significance of the number of nerves affected, clinical form, and education in predicting G2D, suggesting that these features should be prioritized in risk assessment and diagnostic algorithms. However, notable regional disparities were observed. In the midwest, education level had a more pronounced impact on late diagnosis, whereas in the northeast, operational classification and clinical form were the strongest predictors. These variations align with known epidemiological patterns and socioeconomic disparities across Brazilian regions, where access to healthcare services, health literacy, and disease awareness levels differ significantly [39]. Studies have demonstrated that educational attainment is a significant determinant of health-seeking behavior, particularly in neglected tropical diseases [23,32,40]. The heightened impact of education level in the midwest region may reflect disparities in health literacy and healthcare accessibility, further emphasizing the need for targeted awareness and screening programs in this area. The demonstrated regional variations in model performance and feature importance suggest that a one-size-fits-all approach may not be optimal for disease surveillance. Instead, leveraging region-specific models tailored to local epidemiological and health system contexts may enhance the effectiveness of ML-based early detection strategies.

This study has limitations inherent to the limitations of secondary data from the Health Information Systems (HISs), as well as the definition of time to diagnosis of leprosy, with the use of the variable “presence of G2D at the time of diagnosis” as a marker of late diagnosis. On the other hand, the large number of people analyzed, the geographical coverage of the entire national territory, and the growing improvement of the HISs in Brazil add robustness to the analyses, allowing the use of multivariable techniques with adequate statistical power. Data inconsistencies, underreporting, and misclassification may have influenced our results [41]. The reliance on registry-based disability grading limits the assessment of subclinical nerve damage and its progression. Despite these challenges, our use of machine learning methods mitigated some of these limitations by handling missing data more effectively and identifying complex relationships between predictors. Furthermore, the large sample size and nationwide scope enhances the generalizability of our findings.

Despite these limitations, this study has important implications for health services and policy. The increasing trend of G2D highlights the urgent need for improved leprosy control strategies. Our findings suggest several key areas for intervention: (1) scaling up active case finding, particularly contact tracing; (2) improving diagnostic capacity and awareness among primary care providers; (3) addressing patient-related barriers to early diagnosis, such as stigma and lack of health literacy; and (4) integrating machine learning tools into surveillance systems to identify high-risk individuals and target interventions. By addressing these issues, Brazil can move closer to achieving national and international leprosy control targets and reducing the burden of preventable disability.

## 5. Conclusions

This study demonstrates that machine learning models, particularly ensemble approaches, can effectively predict late leprosy diagnosis (G2D) using sociodemographic and clinical variables. The findings highlight persistent and increased diagnostic delays and their association with socioeconomic disparities, underscoring the need for improved surveillance strategies. Integrating predictive modeling into health systems could enhance early detection efforts and reduce the burden of disability in leprosy patients. The observed heterogeneity in feature importance and model performance highlights the need for adaptive strategies in leprosy surveillance, where risk prediction tools should be calibrated according to local disease burden, healthcare infrastructure, and socioeconomic determinants. Future research should explore the implementation of machine learning-based risk stratification tools in real-world settings to improve leprosy control programs.

## Figures and Tables

**Table 1:** Characteristics of leprosy cases assessed for physical disabilities, 2018–2022.

	Grade 2 Physical Disability			
	No	Yes	*p*-Value ^1^	Total
Variable	*n* (%)	*n* (%)		*n* (%)
Sex			<0.001	
Female	56,000 (44.4)	4336 (29.3)		60,335 (42.8)
Male	70,110 (55.6)	10,456 (70.7)		80,566 (57.2)
Age group (in years)			<0.001	
0**–**14	6361 (5.1)	255 (1.7)		6616 (4.7)
15**–**29	18,573 (14.7)	1550 (10.5)		20,123 (14.3)
30**–**49	45,566 (36.1)	4498 (30.4)		50,064 (35. 5)
50**–**69	43,635 (34.6)	6078 (41.1)		49,713 (35.3)
70**–**79	9135 (7.2)	1716 (11.6)		10,851 (7.7)
≥80	2846 (2.3)	696 (4.7)		3542 (2.5)
Race or skin color			<0.001	
White	28,657 (22.9)	3554 (24.3)		32,211 (23.1)
Yellow	1413 (1.1)	150 (1.0)		1563 (1.1)
Brown	75,629 (60.5)	8464 (57.8)		84,093 (60.2)
Black	15,686 (12.6)	2039 (13.9)		17,725 (12.7)
Indigenous	593 (0.5)	77 (0.5)		670 (0.5)
Ignored	3023 (2.4)	349 (2.4)		3372 (2.4)
Education			<0.001	
Higher education (incomplete or complete)	9279 (8.0)	1764 (13.0)		11,043 (8.5)
High school (incomplete or complete)	30,827 (26.5)	4133 (30.5)		34,960 (26.9)
8th grade complete	17,794 (15.3)	2049 (15.1)		19,843 (15.2)
5th to 8th grade incomplete	7959 (6.8)	968 (7.1)		8927 (6.9)
1st to 4th grade (incomplete or complete)	27,056 (23.3)	2266 (16.7)		29,322 (22.5)
Illiterate	7458 (6.4)	463 (3.4)		7921 (6.1)
Ignored and not applicable (under 7 years old)	16,165 (13.9)	1915 (14.1)		18,080 (13.9)
Number of nerves affected			<0.001	
0	54,705 (43.4)	1304 (8.8)		56,009 (39.7)
1	14,421 (11.4)	1308 (8.8)		15,729 (11.2)
2**–**5	37,102 (29.4)	8046 (54.4)		45,148 (32.0)
>5	19,888 (15.8)	4135 (28.0)		24,023 (17.1)
Clinical form			<0.001	
Undetermined	13,864 (11.3)	299 (2.1)		14,163 (10.3)
Tuberculoid	13,762 (11.2)	476 (3.3)		14,238 (10.4)
Virchowian	20,772 (16.9)	4751 (33.0)		25,523 (18.6)
Diforma	66,464 (54.2)	7989 (55.5)		74,453 (54.3)
Not classified	7820 (6.4)	886 (6.1)		8706 (6.4)
Operational classification			<0.001	
Paucibacillary	24,762 (19.6)	468 (3.2)		25,230 (17.9)
Multibacillary	101,342 (80.4)	14,325 (96.4)		11,5667 (82.1)
Case detection mode			<0.001	
Spontaneous demand	40,410 (39.7)	3629 (35.4)		44,039 (39.3)
Referral	44,932 (44.2)	5233 (51.0)		50,165 (44.8)
Collective examination	3660 (3.6)	330 (3.2)		3990 (3.6)
Contact examination	10,104 (9.9)	697 (6.8)		10,801 (9.7)
Other modes	2204 (2.2)	345 (3.4)		2549 (2.3)
Ignored	388 (0.4)	30 (0.3)		418 (0.4)

^1^ Statistical difference among grade 2 physical disability groups (yes/no) were tested with χ2 test for categorical predictors.

**Table 2:** Performance metrics of machine learning models in grade 2 physical disability (G2D) and selected sociodemographic and clinical variables, Brazil, 2018–2022.

	North						
Model	Accuracy	AUC_ROC	Recall	Specificity	Precision	F1 Score	MCC
Random Forest	0.84	0.92	0.91	0.76	0.80	0.85	0.69
LightGBM	0.84	0.93	0.90	0.78	0.80	0.85	0.69
CatBoost	0.83	0.92	0.87	0.79	0.80	0.84	0.66
XGBoost	0.83	0.92	0.88	0.78	0.80	0.84	0.66
Ensemble model	0.84	0.93	0.90	0.78	0.80	0.85	0.69
	Northeast						
	Accuracy	AUC_ROC	Recall	Specificity	Precision	F1 Score	MCC
Random Forest	0.85	0.93	0.90	0.80	0.82	0.86	0.70
LightGBM	0.85	0.93	0.88	0.82	0.83	0.85	0.70
CatBoost	0.84	0.92	0.87	0.81	0.82	0.85	0.68
XGBoost	0.84	0.92	0.88	0.81	0.82	0.85	0.69
Ensemble model	0.85	0.93	0.88	0.82	0.83	0.85	0.70
	Southeast						
	Accuracy	AUC_ROC	Recall	Specificity	Precision	F1 Score	MCC
Random Forest	0.83	0.91	0.89	0.78	0.80	0.84	0.67
LightGBM	0.84	0.92	0.88	0.80	0.81	0.85	0.68
CatBoost	0.83	0.91	0.86	0.80	0.81	0.83	0.65
XGBoost	0.83	0.91	0.87	0.79	0.80	0.83	0.66
Ensemble model	0.84	0.92	0.88	0.80	0.82	0.85	0.69
	South						
	Accuracy	AUC_ROC	Recall	Specificity	Precision	F1 Score	MCC
Random Forest	0.85	0.92	0.89	0.81	0.83	0.86	0.70
LightGBM	0.85	0.93	0.89	0.82	0.83	0.86	0.71
CatBoost	0.86	0.93	0.91	0.81	0.83	0.86	0.72
XGBoost	0.86	0.93	0.90	0.81	0.83	0.86	0.72
Ensemble model	0.86	0.94	0.90	0.82	0.84	0.87	0.73
	Midwest						
	Accuracy	AUC_ROC	Recall	Specificity	Precision	F1 Score	MCC
Random Forest	0.81	0.90	0.87	0.76	0.78	0.82	0.63
LightGBM	0.82	0.91	0.85	0.78	0.80	0.82	0.63
CatBoost	0.81	0.90	0.83	0.79	0.79	0.81	0.62
XGBoost	0.80	0.89	0.82	0.78	0.79	0.81	0.60
Ensemble model	0.81	0.90	0.84	0.78	0.80	0.82	0.63

Abbreviations: XGBoost: eXtreme Gradient Boosting; AUC_ROC: area under the receiver operating characteristic curve; and MCC: Mathew’s correlation coefficient.

## Data Availability

The data used for the investigation were taken from a public database, which can be accessed by any citizen through the DATASUS website at https://datasus.saude.gov.br accessed on 3 February 2025.

## Abbreviations

The following abbreviations are used in this manuscript:

G2D	Grade 2 physical disability
ROC	Receiver Operating Characteristic
AUC-ROC	Area under the ROC curve
SINAN	Notifiable Diseases Information System
MCC	Matthew’s correlation coefficient
HIS	Health Information System
ML	Machine learning
WHO	World Health Organization
